# Supplementary material for: Combined Genome-Wide Association Study and Haplotype Analysis Identifies Candidate Genes Affecting Growth Traits of Inner Mongolian Cashmere Goats
Source: Vet Sci. 2024 Sep 12;11(9):428. doi: 10.3390/vetsci11090428 (PMC11435611; doi:10.3390/vetsci11090428)
Supplement: Supplementary file 1 [file vetsci-11-00428-s001.zip › Supplementary material.pdf]

Combined Genome-wide association study and Haplotype analysis identifies candidate genes affecting growth traits of Inner Mongolian cashmere goats

Xiaofang Ao[1] , Youjun Rong[1] , Mingxuan Han[1] , Xinle Wang[1] , Qincheng Xia[1] , Fangzheng Shang[1], Yan Liu[2], Qi Lv[1], Zhiying Wang[1], Rui Su[1], Yanjun Zhang[1,2,3*] and Ruijun Wang[1*]

Supplementary Materials:

Table S1: Annotation results of significantly associated SNPs of growth traits in IMCGs;

Table S2: Population genetic parameters of related SNPs associated with growth traits in IMCGs;

Table S3: Haplotype analysis of SNPs associated with growth traits of IMCGs;

Table S4: Association analysis between haplotype combination and growth traits of IMCGs.

Figure S1: Phenotypic frequency distribution of growth traits in IMCGs. Body Height (BH), Body Length (BL), Chest Circumference (CC), Chest Depth (CD), Chest Width (CW), Tube Circumference (TC), and Body Weight (BW). The unit of measurement is cm;

Figure S2: Results of linkage analysis disequilibrium and haplotype of SNPs significantly associated with growth traits in IMCGs. The numbers in the grid represent the values of D ‘and r2, and the darker the color, the greater the linkage imbalance between SNPs.
