# Supplementary figures and images for: Combined Genome-Wide Association Study and Haplotype Analysis Identifies Candidate Genes Affecting Growth Traits of Inner Mongolian Cashmere Goats
Source: Vet Sci. 2024 Sep 12;11(9):428. doi: 10.3390/vetsci11090428 (PMC11435611; doi:10.3390/vetsci11090428)

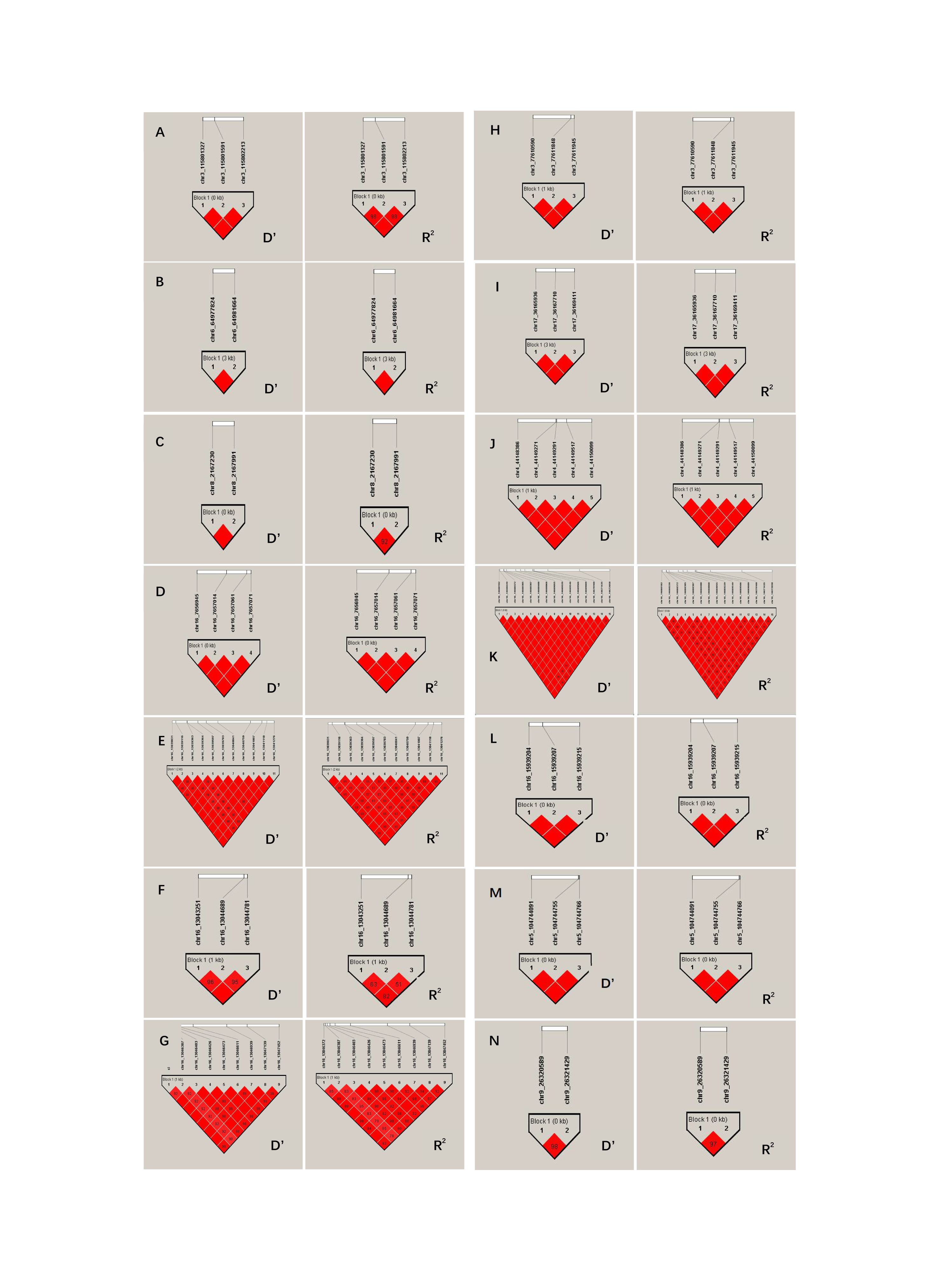

Supplement: Supplementary file 1 [file vetsci-11-00428-s001.zip › Supplementary figure/Figure s1-2/Fig S2-1.tif]

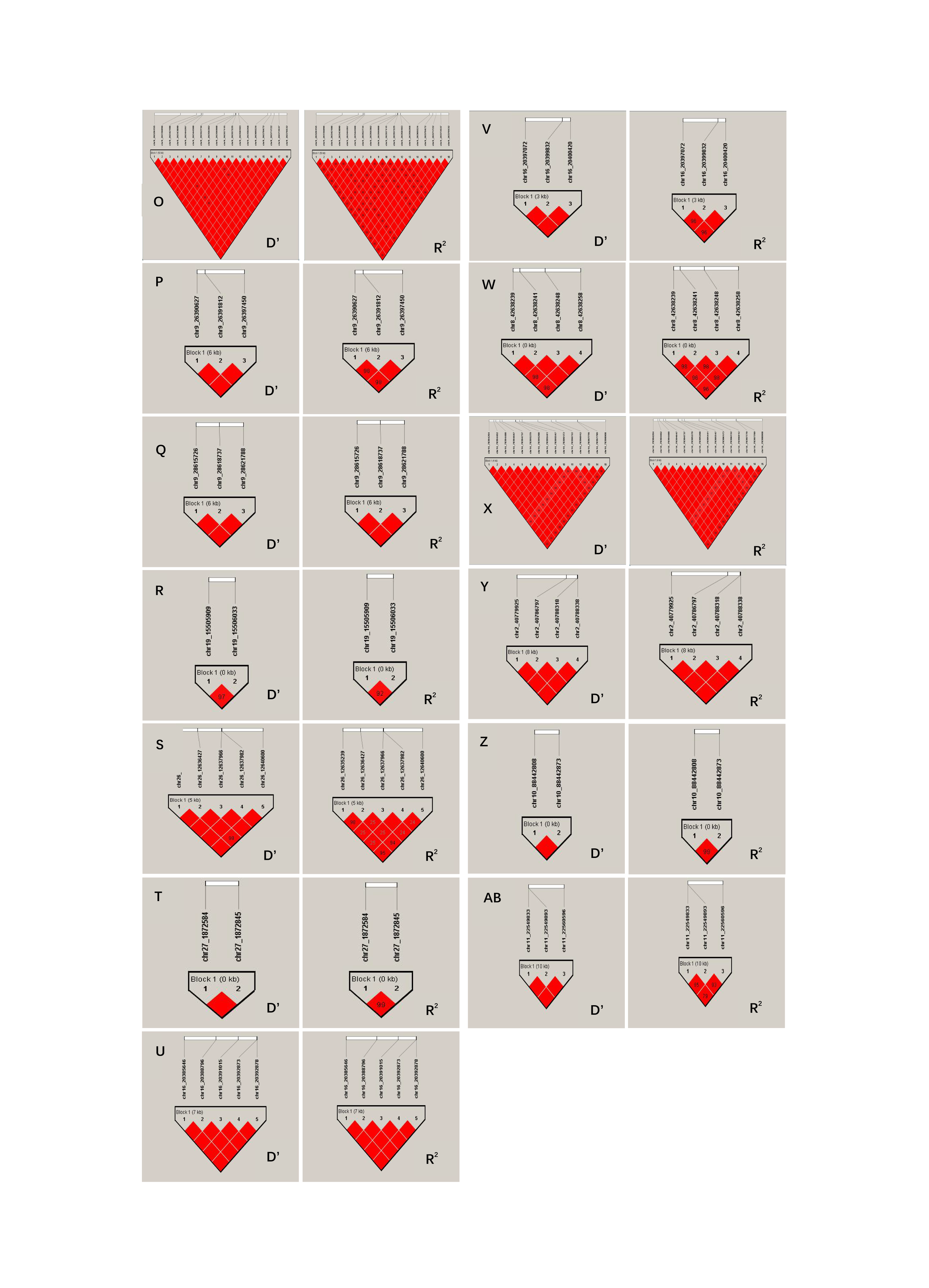

Supplement: Supplementary file 1 [file vetsci-11-00428-s001.zip › Supplementary figure/Figure s1-2/Fig S2-2.tif]
